# Essential role of Card11 in airway hyperresponsiveness in high-fat diet-induced obese mice

**DOI:** 10.1038/s12276-024-01367-z

**Published:** 2024-12-13

**Authors:** Hyun-Seung Lee, Byung-Keun Kim, Suh-Young Lee, Hyuktae Kwon, Heung-Woo Park

**Affiliations:** 1 Biomedical Research Institute, Seoul National University Hospital, Seoul, Republic of Korea; 2 Department of Internal Medicine, Korea University College of Medicine, Seoul, Republic of Korea; 3 Department of Internal Medicine, Seoul National University Hospital, Seoul, Republic of Korea; 4 Department of Family Medicine, Seoul National University Hospital, Seoul, Republic of Korea; 5 Department of Internal Medicine, Seoul National University College of Medicine, Seoul, Republic of Korea

**Keywords:** Innate immunity, Translational research

## Abstract

A high-fat diet (HFD) can induce airway hyperresponsiveness (AHR) in obese mice, independent of allergic sensitization. This study aimed to identify the key molecules related to AHR in HFD-induced obese mice. In a cluster analysis of time series gene expression in the adipose and lung tissues of HFD-induced obese mice, we identified the Caspase Recruitment Domain Family Member 11 (*Card11*) gene as an essential molecule. We measured CARD11 expression in peripheral blood mononuclear cells (PBMCs) from obese individuals with asthma and performed Card11 signal inhibition in HFD-induced obese mice via *Card11* siRNA. Card11 expression was significantly increased in M1 macrophages (IL-1β^+^CD11c^+^CD206^-^ in CD11b^+^) in adipose tissue and in ILC3s (RORγt^+^ in IL7R^+^ of Lin^-^) in lung tissue from HFD-induced obese mice. In addition, CARD11^+^ populations among ILC3s and LPS-stimulated IL-1β^+^CD16^+^ monocytes from the PBMCs of obese individuals with asthma were significantly greater than those from obese controls or nonobese individuals with asthma. AHR in HFD-induced obese mice disappeared when we inhibited the Card11 signaling pathway by administering *Card11* siRNA during the first or last seven weeks of the 13-week HFD feeding. Finally, we confirmed that *Card11* siRNA decreased the number of M1 macrophages in adipose tissue and the number of ILC3s in lung tissue in vitro. Card11 significantly contributes to the development of AHR in HFD-induced obese mice by affecting immune cells in both adipose and lung tissues. The middle stage of HFD feeding seemed to be critical for these processes.

## CARD11 drives asthma response in obese mice models

Obesity is a metabolic disease and a major risk factor for several non-communicable diseases. The link between obesity and asthma is not fully understood. The study used a murine model to investigate how obesity affects asthma. Researchers fed mice a high-fat diet and analyzed gene expression in their adipose and lung tissues. They identified the Card11 gene as a key player in airway hyperresponsiveness in obese mice. This was an experimental study involving mice. Results showed that Card11 expression increased in immune cells from both adipose and lung tissues of obese mice. Inhibiting Card11 reduced AHR and inflammation. Researchers concluded that Card11 is crucial in obesity-related asthma. Future studies could explore Card11 as a therapeutic target for asthma in obese individuals. This summary was initially drafted using artificial intelligence, then revised and fact-checked by the author.

## Introduction

Obesity is a metabolic disease and a major risk factor for several noncommunicable diseases. Since the first epidemiological link between asthma and obesity was reported by Camargo et al.^1^, substantial evidence has been accumulated suggesting possible associations between obesity and asthma^2,3^. A murine model of obesity induced by a high-fat diet (HFD) is widely utilized for studying the relationship between obesity and asthma. For example, Kim et al. reported that upregulation of the NLRP3 inflammasome and IL-1β is implicated in obesity-associated airway hyperresponsiveness (AHR) in this murine model^4^. A HFD itself can induce AHR in mice, independent of allergic sensitization^5^. Currently, systemic inflammation is considered a factor bridging increased adipose tissue and hyperreactive airways^3,6^. These findings suggest that AHR in HFD-induced obese mice needs to be explored from the perspective of interactions between adipose and lung tissues. However, the detailed mechanisms underlying HFD-induced AHR, especially the sequential interactions between adipose and lung tissues, are not fully understood.

In this study, we aimed to identify the key molecules associated with AHR in HFD-induced obese mice. First, we performed a cluster analysis of time series gene expression in adipose and lung tissues obtained from HFD-induced obese mice to identify important genes connecting the two tissues, resulting in AHR. As gene expression is a tightly regulated spatiotemporal process, an analysis of time series gene expression provides a unique window for understanding its pathogenesis^7,8^. Through this analysis, we identified the Caspase Recruitment Domain Family Member 11 (*Card11*) gene. Card11, a scaffold protein that combines with the Bcl10 and Malt1 proteins to form the Card11/Bcl10/Malt1 signalosome^9^, is involved mainly in the signaling of blood cells such as B/T lymphocytes, natural killer cells, and mast cells, and plays an important role in inflammatory gene expression through the activation of the NF-κB/MAPK pathway in various inflammatory diseases^10–12^. In addition, Card11 signaling upregulates the alanine serine cysteine transporter 2, which transports glutamine and stimulates mTORC1 to promote Th1/Th17 polarization^13^. HFD-induced AHR was previously reported to be independent of adaptive immunity but dependent on innate lymphocyte cell type 3 (ILC3) producing IL-17^4^. Moreover, ~55% of patients with allergic asthma have hypomorphic dominant-negative *CARD11* mutations^14^. On the basis of these findings, we selected *Card11* as a target gene in this study.

To evaluate its clinical relevance, we measured CARD11 expression in ILC3 and CD16^+^ cells obtained from the peripheral blood mononuclear cells (PBMCs) of obese/nonobese individuals with asthma and their corresponding controls. As mentioned previously, the increase of ILC3 in lung tissue due to the increase in macrophage type 1 (M1) in adipose tissue is the main mechanism of HFD-induced AHR^4^. Adipose tissue is a key site for the chronic and systemic inflammation observed in obese mice and humans^15^, and activated circulating immune cells such as monocytes, migrate to inflamed adipose tissue and differentiate into macrophages^16^. Interestingly, an increase in the number of circulating CD16^+^ monocytes was observed in obese humans compared with lean humans and this increase was significantly attenuated after weight loss^17,18^. On the basis of these observations, we assumed that ILC3 and CD16^+^ cells in the blood are important cells linking adipose and lung tissue in the development of AHR. Finally, we inhibited Card11 signaling in HFD-induced obese mice via *Card11* siRNA to assess the role of Card11 in obesity-associated AHR.

## Materials and methods

All mouse experiments were approved by the Institutional Animal Care and Use Committee of the Institute of Laboratory Animal Resources at Seoul National University (SNU-190922-2 and IACUC 23-0208-S1A0). The study protocol was approved by the Institutional Review Board of the Seoul National University Hospital (H-2107-149-1237), and informed consent was obtained from all participants.

### Cluster analysis of time series gene expression

Four-week-old male C57BL/6 mice were purchased from Koatech, Inc. (Gyeonggi-do, Korea). The obese mice were fed a HFD (60 kcal%, D12492; Research Diets, Inc., New Brunswick, NJ, USA), whereas the control mice were fed a standard chow diet (3 kcal% fat). The HFD-fed (HFD group) and control (Chow group) mice were sacrificed at 4, 7, 10, and 13 weeks (four mice per time point in both groups) and the left lung and epididymal white adipose tissue were collected for gene expression analysis. To evaluate AHR, the mice were challenged with increasing doses of inhaled methacholine (0, 12.5, 25, 50, and 100 mg/mL; Sigma‒Aldrich, St. Louis, MO, USA), and airway resistance (cmH2O/mL/s) was measured via a Flexivent system (Scireq, Montreal, Canada)^19^. We chose a value of 100 mg/mL as a representative result^20^. After the adipose and lung tissues were homogenized, total RNA was extracted using TRIzol reagent (Invitrogen Life Technologies, Grand Island, NY, USA) according to the manufacturer’s instructions. DNase digestion was used to remove any DNA contamination, and the RNA was reprecipitated in ethanol to ensure that there was no phenol contamination. For quality control, the RNA purity and integrity were evaluated using an Agilent 2100 Bioanalyzer (Agilent Technologies, Palo Alto, CA, USA). Microarray analysis was performed on 100 ng of total RNA samples using the GeneChip™ Mouse Genome 430 2.0 Array (Affymetrix, Foster City, CA, USA). The generation of labeled cDNA and processing of the array were performed according to the Affymetrix standard protocol. After staining, the intensities were determined with a GeneChip scanner 3000 (Affymetrix) controlled by GCOS Affymetrix software. A cluster analysis of time-series gene expression in 32 samples (four time points, two dietary methods, and four mice per time point) was performed via the package maSigPro in R^21^. maSigPro fits an optimized polynomial linear model to describe the dynamics of gene expression under one or more experimental conditions and selects genes with significant model coefficients^21^. The package incorporates a clustering function to visualize genes with similar profiles. All analyses were performed using R version 4.0.3 software. To assign biological meaning to genes in each cluster, we evaluated the function of each gene using the “Human‒Mouse: Disease Connection” tool implemented in “Mouse Genome Informatics” (http://www.informatics.jax.org/).^22^ This tool provides phenotypes directly attributed to mutations of mouse genes. We focused on phenotypes associated with immunological or respiratory system changes.

### Evaluation of CARD11 expression in human PBMCs

We evaluated the expression of CARD11 (a key molecule identified from a cluster analysis of time series gene expression) in PBMCs from four human groups: obese controls, nonobese controls, obese individuals with asthma, and nonobese individuals with asthma. As CARD11 is involved mainly in signaling in blood cells^10–12^, PBMCs were selected for this expression analysis. Obesity was defined as a body mass index ≥25 kg/m^2^ according to the Asia-Pacific classification^23^. The detailed phenotyping methods are presented in Supplementary Material. PBMCs were prepared from buffy coats via density gradient centrifugation using Ficoll‒Paque Plus (GE Healthcare, Chicago, IL, USA). The cells were stored in freezing solution at −196 °C before the experiment. Heparinized peripheral blood was layered over an equal volume of Histopaque 1077 (Sigma‒Aldrich) and centrifuged according to the manufacturer’s instructions. Mononuclear cells were collected from the interface between the histopaque and the plasma. After washing, the PBMCs (5 × 10^6^ cells) were preincubated with Human TruStain FcX^TM^ (Fc Receptor Blocking Solution, BioLegend 422301; BioLegend, San Diego, CA, USA; 1:50) and stained with antibodies against lineage markers^24^ (CD3, CD14, CD16, CD19, CD20 and CD56; Lineage marker kit; BioLegend), FITC-conjugated anti-human CD11c (BioLegend), FITC-conjugated anti-human CD123 (BioLegend), PE/cy7-conjugated anti-human CD127 (BioLegend), APC-conjugated anti-human CD117 (BioLegend), and BV711-conjugated anti-human NKp44 (BD Biosciences, Franklin Lakes, NJ, USA). ILC3s were defined as RORγt^+^Lin^–^CD127^+^CD161^+^CD117^+^NKp44^+^^25^. For intracellular staining, the cells were permeabilized (Cytofix/Cytoperm Kit; BD Biosciences) and incubated with PE-conjugated anti-RORrγt (BD Biosciences) and AF594-conjugated anti-CARD11 antibodies (Santa Cruz Biotechnology, Santa Cruz, CA, USA). At least 400,000 cells were read via BD LSRFortessa™ (BD Biosciences), and the results were analyzed using FlowJo software (BD Biosciences). PBMCs (5 × 10^5^ cells/well) were seeded in a 96-well culture plate and stimulated with 50 ng/ml lipopolysaccharide (LPS)^26^ (*Escherichia coli* 0111:B4; Sigma‒Aldrich) overnight. The cells were also treated with GolgiStop (BD Bioscience) and stained with an Alexa 700-conjugated anti-CD16 (BioLegend) antibody. For intracellular staining, the cells were permeabilized and incubated with FITC-conjugated anti-IL-1β (BioLegend) and AF594-conjugated anti-CARD11 antibodies. Information on the antibodies used for flow cytometry, including the dilution factors, is presented in Supplementary Table 1.

### Evaluating the role of Card11 in the development of AHR in HFD-induced obese mice

We first evaluated the expression of Card11 in RORγt-expressing ILC3s and M1 macrophages from the HFD and Chow groups at each time point since these two groups of cells were reported to mediate obesity-associated AHR^4^. We assessed the role of Card11 by administering *Card11* siRNA^27^ to mice at different time points during HFD feeding. In brief, 0.45 mg/kg *Card11* siRNA (sc-44937; Santa Cruz Biotechnology) was reconstituted in RNase-free water and mixed with an equal volume of siPORT™ Transfection Agent (AM4511; Thermo Fisher Scientific, Santa Clara, CA, USA). *Card11* siRNA and a control sequence (scrambled siRNA) were administered intraperitoneally. Flow cytometry was performed using an AF594-CARD11 antibody (Santa Cruz Biotechnology) to assess the silencing effects.

### Cell analysis of mouse lung and adipose tissues by flow cytometry

For single lung cell preparation, lung tissue (in RPMI) was obtained, minced into small pieces, placed in RPMI medium containing 1.6 mg/mL collagenase type 4 (Worthington, Lakewood, NJ, USA), and digested at 37 °C. After filtration (40 µm) with RPMI and centrifugation, RBC lysis buffer (BioLegend) was used to remove RBC from the whole cells. After single-cell preparation, the cells were stimulated with PMA (0.1 µg/mL), ionomycin (1 µg/mL) and GolgiStop. For single adipose cell preparation, WAT (in HBSS plus 1% penicillin/streptomycin) was obtained; after centrifugation, the upper layer of adipose tissue was minced into small pieces, placed in HBSS medium containing 2 mg/mL collagenase type 4, and digested at 37 °C^28^. After filtration (70 µm) with DMEM and centrifugation, RBC lysis buffer was used to remove RBC from the whole cells^29^. We analyzed ILC3s from mouse lung tissue and M1 macrophages from mouse WAT. For fluorescence-activated sorting of these cells, the following antibodies were used: APC-conjugated anti-RORγt (Thermo Fisher Scientific), PE/Cy7-conjugated anti-pro-IL-1β (Thermo Fisher Scientific), anti-CD206 (R and D Systems, Minneapolis, MN, USA), AF647-conjugated anti-goat IgG (Thermo Fisher Scientific), BV711-conjugated anti CD4 (BioLegend), BV421-conjugated anti-IL-17A (BioLegend), FITC-conjugated anti-CD11b (BioLegend), and APC/Cy7- conjugated anti-CD11c (BD Biosciences) antibodies. For ILCs, lineage-negative cells (Lin^–^), CD3^–^, CD4^–^, CD8^–^, CD11b^–^, CD11c^–^, CD19^–^, F4/80^–^, FcεRI^–^, and CD49b^–^, were sorted using CD127 as a surface marker^30^. ILC3s were defined as Lin^−^CD127^+^RORγt^+^ lymphoid cell populations. M1s were gated for CD11b^+^CD206^–^CD11c^+^. Next, we detected the expression of pro-IL-1β in M1 macrophages from adipose tissue. The antibodies were titrated prior to use in the experiments, and species-specific fluorescence-conjugated isotype control antibodies were used to identify background isotype binding. Information on the antibodies used for flow cytometry, including the dilution factors, is presented in Supplementary Table 1. Flow cytometry was performed using a BD LSRFortessa™, and the results were analyzed using FlowJo software (BD Biosciences).

### Histopathology

The left lung and adipose tissue of the mice were fixed in 10% neutral buffered formalin and embedded in paraffin, and 3 mm sections were stained with hematoxylin and eosin (H&E, Sigma‒Aldrich). The sectioned lung tissue was stained with Masson’s trichrome (Abcam, Cambridge, UK) to evaluate collagen deposition. Staining was performed according to the manufacturer’s protocols and stained images were captured using a digital image program (iSolution Lite; IMT i-solution Inc., Vancouver, Canada) with a Nikon light microscope.

### Evaluating the effects of in vitro Card11 inhibition on ILC3 differentiation from ILCs in naïve mouse lungs and M1 macrophage induction from BMDMs

To gain further insight, we examined the in vitro effects of Card11 inhibition of IL-17 producing ILC3s differentiated from ILCs in naïve mouse lungs and on M1 macrophage induction from bone marrow-derived macrophages (BMDMs). ILCs (5 × 10^4^ cells/well), which were gated as CD45^+^Lin^–^CD127^+^ cells from the lung tissue of naïve mice (n = 12), were stimulated with IL-2 (10 ng/mL; R and D Systems), IL-7 (10 ng/mL; R and D Systems), IL-1β (20 ng/mL; BioLgend), and IL-23 (20 ng/mL; Thermo Fisher Scientific) for 10 days to induce ILC3 differentiation^29^. ILC3s were treated with *Card11* siRNA (100 nmol/L)^31^ or control siRNA with siPORT™ Transfection Agent (3 μL/well), and after 5 h, the cells were washed and then incubated overnight. ILC3s were stimulated with PMA (100 ng/mL), ionomycin (1 μg/mL), and GolgiStop 4 h before cell harvesting, and were then stained with PE/Cy7-conjugated anti-IL-7R (BioLegend). Finally, the cells were permeabilized using a Cytofix/Cytoperm kit (BD Biosciences) and incubated with APC-conjugated anti-RORγt (Thermo Fisher Scientific) and BV421-conjugated anti-IL-17A (BioLegend) antibodies. BMDMs from a 6-week-old male C57BL/6 naïve mouse were prepared using previously described methods^32^. Briefly, the isolated bone marrow cells were resuspended (2 × 10^6^ cells/mL) in BMDM growth medium (containing 10 ng/mL monocyte-colony stimulating factor) and seeded in T75 culture flasks, and fresh BMDM growth medium was replaced on day 3. On day 7, mature BMDMs were seeded in six-well culture plates and detected by identifying CD11b^+^ cells and then they were treated with *Card11* siRNA (100 nmol/L) or NC siRNA with siPORT™ Transfection Agent (3 μL/well). After 5 h, the cells were stimulated with 100 ng/mL of LPS (Sigma‒Aldrich) to induce M1 polarization^29^. Each sample was read on a BD LSRFortessa™.

### Statistical analysis

All in vivo and in vitro experiments were performed at least three times. The results are expressed as the means ± standard deviations and P < 0.05 indicates statistical significance. The data were analyzed for significance using two-way analysis and one-way analysis of variance with Bonferroni posttest correction for multiple comparisons. Statistical analyses were performed using GraphPad Prism 8.0.1 software (La Jolla, CA, USA).

## Results

### *Card11* is a key gene associated with AHR in HFD-induced obese mice

Compared with the Chow group, the HFD group had significantly greater body weights and epididymal WAT at all four time points (4, 7, 10, and 13 weeks) (Supplementary Fig. 1a). The HFD group showed a significantly increased AHR only at 13 weeks (Supplementary Fig. 1b). The infiltration of inflammatory cells, collagen deposition in lung tissue, and inflammation in adipose tissue in the HFD group were prominent at 13 weeks (Supplementary Fig. 1c–e). Compared with those in the Chow group, the number of neutrophils (CD11c^–^Gr-1^+^ in CD11b^+^) in the lung tissue of the HFD group significantly increased at 4 weeks, and this increase persisted at all four time points (Supplementary Fig. 1f, g).

**Fig. 1:**
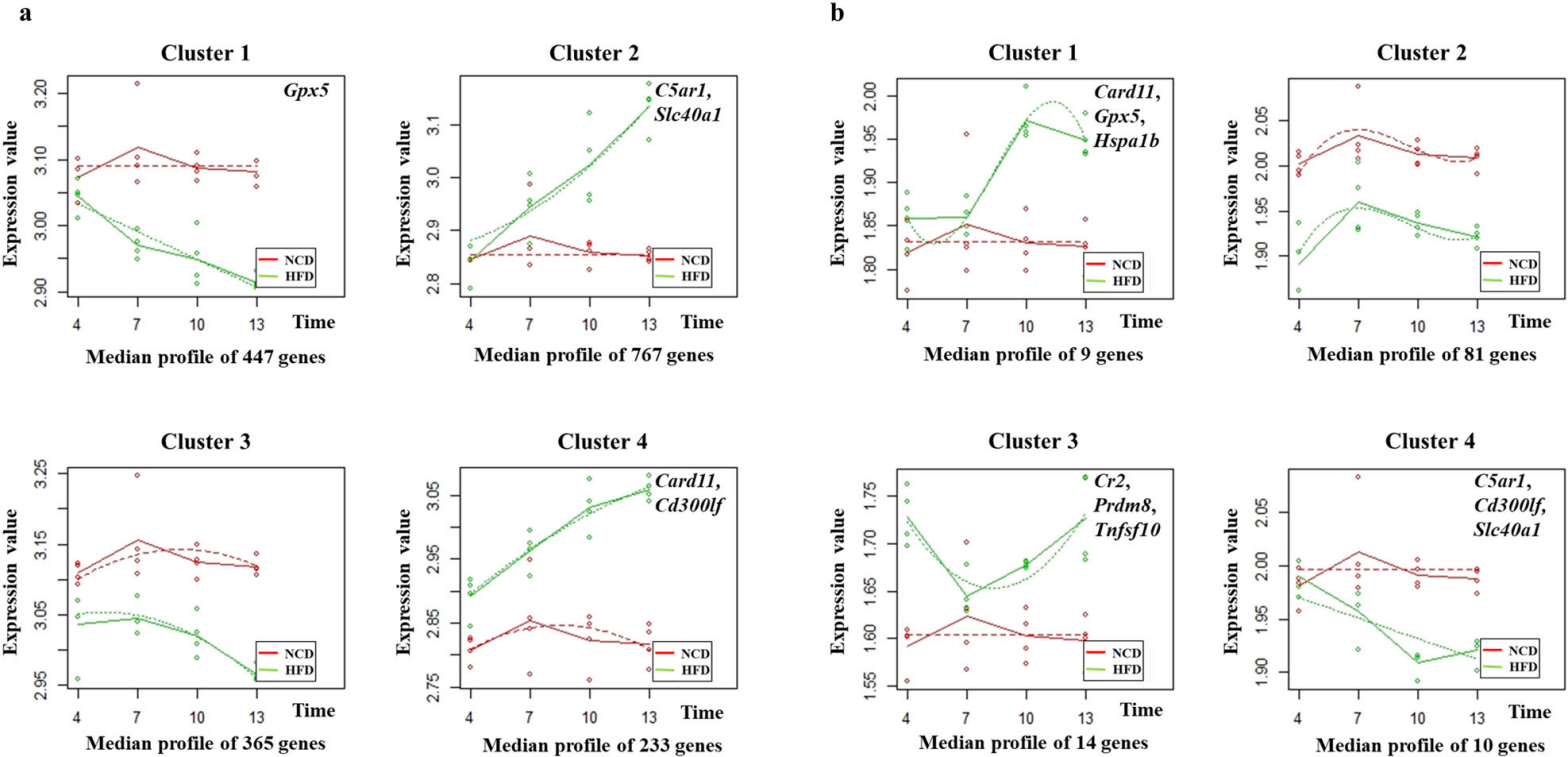
Time series clustering of gene expression in the adipose and lung tissues from HFD-induced obese mice. **a** Adipose tissue. **b** Lung tissue. Gene expression profiles in adipose and lung tissues were grouped into four clusters that presented distinct temporal profiles, and the median expression of all genes in each cluster was plotted for each diet and time. The solid lines connecting the average expression values represent the trends for each diet group, and the dashed lines represent the regression curves fitted to the data. Functionally important genes belonging to 4 clusters (Supplementary Table 3) are presented in each box. Eight mice per group were used. Chow normal chow diet, HFD high-fat diet.

A total of 22,067 genes were evaluated via cluster analysis of time series gene expression. In both adipose and lung tissues, four clusters with different profiles were identified over time (Fig. 1a, b). Supplementary Table 2 lists the genes comprising each cluster. After evaluating the functions of individual genes belonging to the four clusters identified from lung tissue, we selected nine genes (*Card11, Gpx5, Hspa1b, Cr2, Prdm8, Tnfsf10, C5ar1, Cd300lf*, and *Slc40a1*) whose mutations directly cause changes in the immunologic and respiratory phenotypes of mice (Supplementary Table 3). We found that *Card11, Gpx5, C5ar1, Cd300lf*, and *Slc40a* were also assigned to the four clusters identified from adipose tissue (Supplementary Table 3). After sequential evaluation of gene expression changes in adipose and lung tissue and careful reading of previous reports, we selected *Card11* as a key gene associated with AHR in HFD-induced obese mice.

### CARD11 expression is increased in ILC3s and CD16^+^ monocytes from PBMCs of obese individuals with asthma

**Fig. 2:**
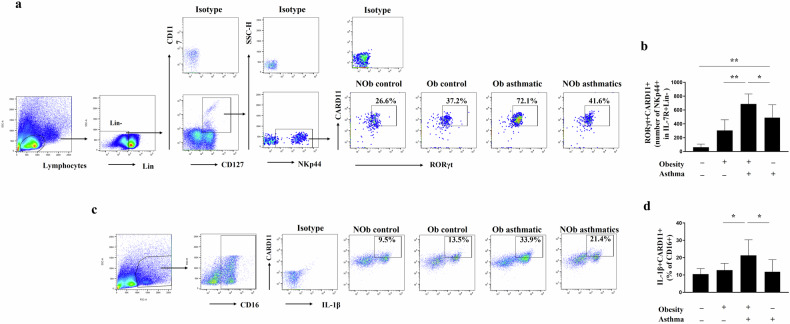
CARD11 expression in ILC3s and CD16^+^ monocytes from human PBMCs. **a**, **b** Representative gating strategy and the total number of CARD11^+^RORγt^+^ in ILC3s (CD45^+^Lin^−^CD127^+^CD161^+^CD117^+^ NKp44^+^) among human PBMCs. The percentage represents the population of CARD11^+^RORγt^+^ in ILC3s. **c**, **d** Representative gating strategy and the frequency of CARD11^+^Pro-IL-1β^+^ cells in CD16^+^ monocytes from LPS-stimulated human PBMCs. The percentage represents the population of CARD11^+^Pro-IL-1β^+^ in CD16^+^ monocytes. Results are presented as means ± standard deviations. ^*^P < 0.05 and ^**^P < 0.01. Statistical analysis was performed via one-way ANOVA with Bonferroni’s multiple comparisons test. Nob nonobese, Ob obese.

The enrolled participants were classified into four groups; obese controls (n = 10), nonobese controls (n = 11), obese individuals with asthma (n = 10), and nonobese individuals with asthma (n = 10). The baseline demographics are presented in Supplementary Table 4. The expression of CARD11 in ILC3s (RORgt^+^CARD11^+^ in NKp44^+^CD117^+^CD127^+^Lin^–^ lymphocyte) in obese individuals with asthma was significantly greater than that in obese controls or nonobese individuals with asthma (Fig. 2a, b). LPS stimulation significantly increased the pro-IL-1β^+^CARD11^+^ population among CD16^+^ cells in obese individuals with asthma compared to that in obese controls or nonobese individuals with asthma (Fig. 2c, d). CD16^+^ monocytes in PBMCs are thought to contribute to chronic inflammation by secreting inflammatory cytokines^33^.

### Card11 expression is increased in ILC3s from the lung tissue of HFD-induced obese mice and M1 macrophages from the adipose tissue of HFD-induced obese mice

**Fig. 3:**
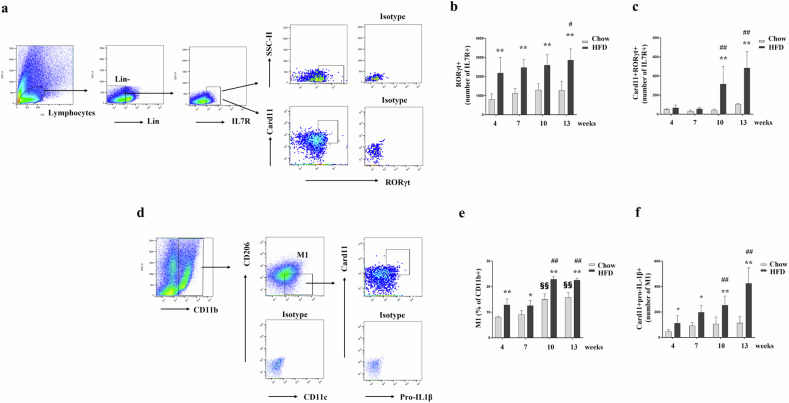
Card11 expression in ILC3s from the lung tissue of HFD-induced obese mice and M1 macrophages from the adipose tissue of HFD-induced obese mice. **a**–**c** Representative gating strategy and the total number of CD127^+^RORγt^+^ ILC3s (**b**) and Card11 in CD127^+^RORγt^+^ ILC3s (**c**) from the lung tissue of each group. **d**–**f** Representative gating strategy and the frequency of M1s (CD11c^+^CD206^−^) in CD11b^+^ cells (**e**) and the total number Card11^+^pro-IL-1β^+^ M1s (**f**) in the adipose tissue of each group. Five animals per group were used. Results are presented as means ± standard deviations. #, differences in the HFD group (^#^P < 0.05 and ^##^P < 0.01; compared with 4 weeks). §, differences in the Chow group (^§§^P < 0.01; compared with 4 weeks). *, differences between the HFD and Chow group (^*^P < 0.05 and ^**^P < 0.01). Statistical analysis was performed using two-way ANOVA with Sidak’s multiple comparisons test. Chow normal chow diet, HFD high-fat diet, Card11 Caspase Recruitment Domain Family Member 11.

Compared with that in the Chow group, the ILC3 population in the lung tissue significantly increased in the HFD group at all four time points (Fig. 3a, b). Moreover, the expression of Card11 in ILC3s from the lung tissue of the HFD group was significantly greater than that in the Chow group at 10 and 13 weeks (Fig. 3c). Similarly, the M1 population in the adipose tissue of the HFD group was significantly greater at all four time points (Fig. 3d, e). Similarly, the expression of Card11 in M1 macrophages from the adipose tissue of the HFD group significantly increased compared with that in the Chow group at all four time points (Fig. 3f).

### Card11 signal inhibition significantly attenuated AHR in HFD-induced obese mice in a manner dependent on the time of *Card11* siRNA administration

**Fig. 4:**
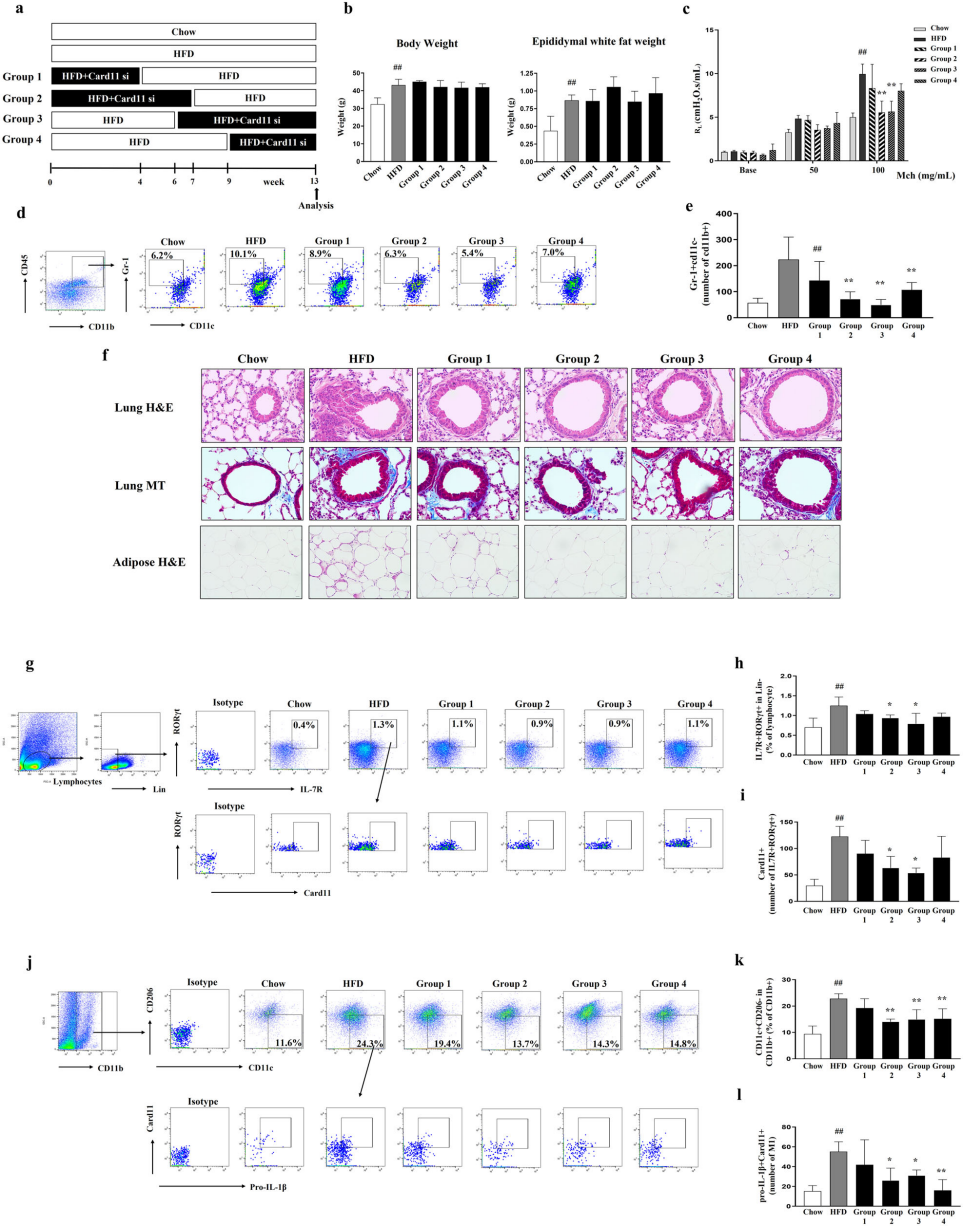
Evaluation of changes in HFD-induced obese mice treated with *Card11* siRNA at each time point. **a** Experimental protocol. **b** Body weight and epididymal white fat weight in each group. **c** Methacholine (Mch)-induced AHR (Mch, mg/ml) in each group (Y-axis represents cmH_2_O/mL/s). **d**, **e** Representative gating strategy and the total number of neutrophils (Gr^–^1^+^CD11c^–^ in CD11b^+^) in the lung tissue. The percentage represents the population of Gr-1^+^CD11c^–^ cells in CD11b^+^ population. **f** Histological examination of lung tissue (H&E stain, × 400; Masson’s trichrome stain, × 400) and adipose tissue (H&E stain, × 200). Scale bars of H&E stained lungs represent 100 µm. Scale bars of lung Masson’s trichrome (MT) staining and adipose H&E staining represent 50 µm. **g**–**i** Representative gating strategy and the frequencies of CD127^+^RORγt^+^ ILC3s cells (**h**) and the total number of Card11 in CD127^+^RORγt^+^ ILC3s (**i**) from the lung tissue in each group. The percentage represents the population of CD127^+^RORγt^+^ ILC3s in lymphocytes. **j**–**l** Representative gating strategy and the frequencies of M1s (CD11c^+^CD206^-^) in CD11b^+^ cells (**k**) and the total number of Card11^+^pro-IL-1β^+^ in M1s (**l**) in the adipose tissue of each group. The percentage represents the population of M1s (CD11c^+^CD206^–^) in CD11b^+^ cells. Five animals per group were used. Results are presented as means ± standard deviations. ^#^P < 0.05 and ^##^P < 0.01 (compared with the Chow group). ^*^P < 0.05 and ^**^P < 0.01 (compared with the HFD group). Statistical analysis was performed using one-way ANOVA with Bonferroni’s multiple comparisons test. Chow, normal chow diet, HFD high-fat diet, si siRNA.

To assess the role of Card11 in the development of AHR in HFD-induced obese mice, we inhibited Card11 signaling via the intraperitoneal administration of *Card11* siRNA and evaluated the changes in phenotypes, including AHR. On the basis of our preliminary experiments evaluating the persistence of the reduction in the CD45^+^Card11^+^ population in lung cells after *Card11* siRNA administration, we selected 72 h as the interval for *Card11* siRNA administration (Supplementary Fig. 2a, b). In addition, we confirmed that control siRNA administered to HFD-induced obese mice at each time point did not result in significant changes in body weight, AHR, neutrophils in lung cells, inflammation in lung and adipose tissue, the ILC3 population in lung tissue, or the M1 population in adipose tissue (Supplementary Fig. 3a–h). As shown in Fig. 1, *Card11* expression in adipose tissue was increased at 4 weeks after the initiation of HFD (Cluster 4), but *Card11* expression in lung tissue started to increase at 7 weeks after the initiation of HFD (Cluster 1). Thus, we varied the timing of *Card11* siRNA administration as follows; during the first 4 weeks (Group 1), first 7 weeks (Group 2), last 7 weeks (Group 3), and last 4 weeks (Group 4) (Fig. 4a). Compared with those in the HFD group, the AHR, neutrophils in the lung cells, inflammation in the lung and adipose tissues, and collagen deposition in the lung tissue in Groups 2 and 3 were significantly lower (Fig. 4b–f). Similarly, the ILC3 population (RORgt^+^ in IL7R^+^ of Lin^−^) in the lung tissue and Card11 expression in ILC3s were significantly lower in Groups 2 and 3 than in the HFD group (Fig. 4g–i). In addition, the proportion of M1 macrophages (CD11c^+^CD206^–^ in CD11b^+^) in the adipose tissue and Card11 expression in IL-1β^+^ M1 macrophages were significantly lower in the Groups 2, 3, and 4 than in the HFD group (Fig. 4j–l).

### Card11 signal inhibition significantly reduced IL-17^+^RORγt^+^ producing ILC3s differentiated from ILCs of naïve mouse lungs and pro-IL-1β^+^ M1 macrophage induction by LPS from BMDMs in vitro

**Fig. 5:**
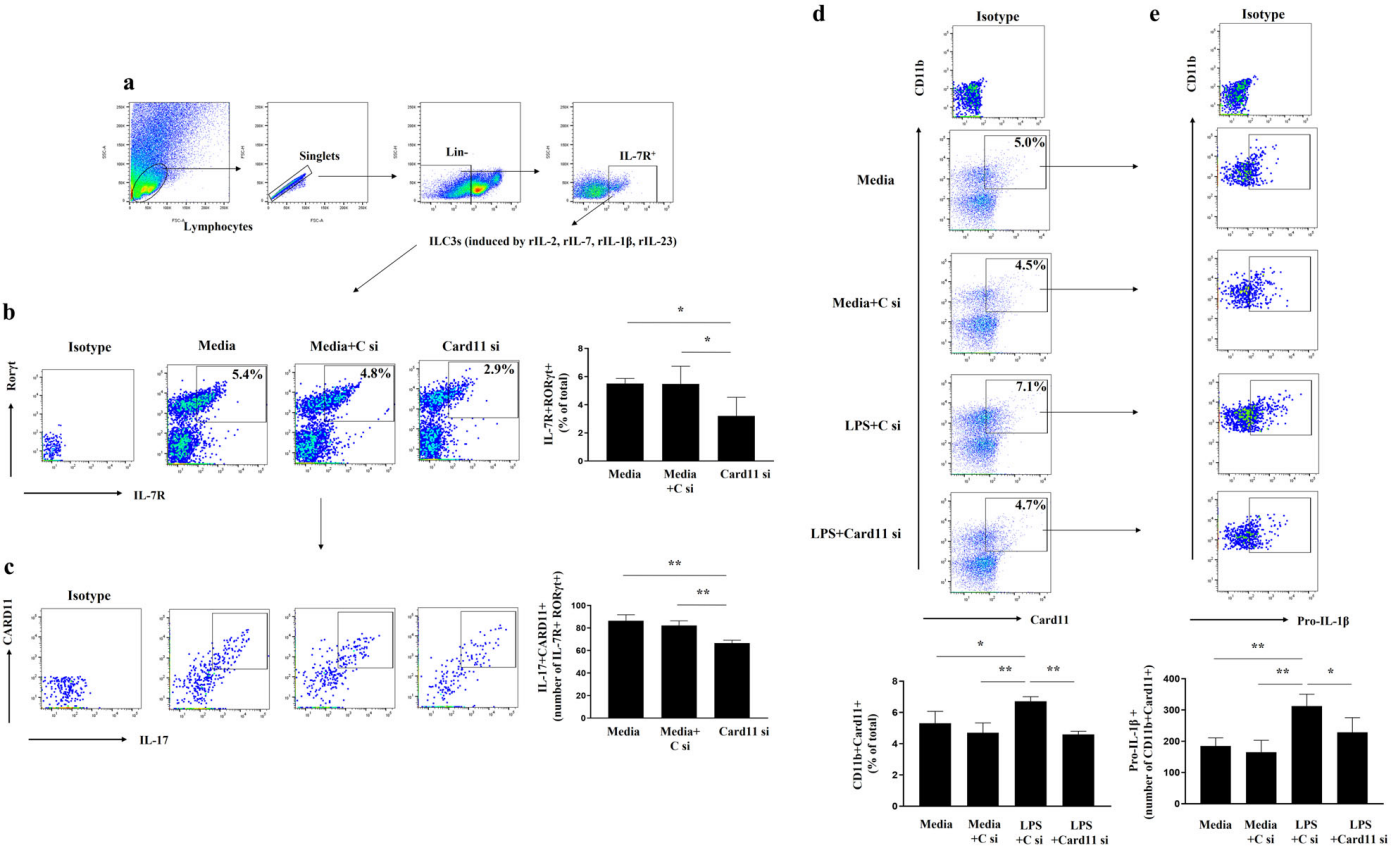
Effects of Card11 signaling inhibition on the IL-17^+^RORγt^+^ producing ILC3s differentiated from ILCs of naïve mouse lungs and the induction of IL-1β^+^ M1 macrophage by LPS from BMDMs in vitro. **a** Representative gating strategy for Lin^-^CD127 ILC3s obtained from naïve mouse lung tissue. Gating plot and the frequencies of IL7R^+^RORγt^+^ ILC3s cells (**b**) and the total number of Card11^+^IL-17^+^ in IL-7R^+^RORγt^+^ ILC3s (**c**) in the lung tissue after culture. The percentage represents the population of IL7R^+^RORγt^+^ ILC3s in total cells. Gating plot and the frequencies of CD11b^+^Card11^+^ cells (**d**) and the total number of Pro-IL-1β^+^ in CD11b^+^Card11^+^ LPS-induced M1 macrophages (**e**) from BMDMs. The percentage represents the population of CD11b^+^Card11^+^ in total cells. Each group of ILC3s and BMDMs consisted of four wells and the experiments were repeated three times. All of the results were similar; thus, the results from a representative experiment are shown. Results are presented as means ± standard deviations. ^*^P < 0.05 and ^**^P < 0.01. Statistical analysis was performed via one-way ANOVA with Bonferroni’s multiple comparisons test. ILC3 type 3 innate lymphoid cell, BMDM bone marrow-derived macrophage, LPS lipopolysaccharide, C si control siRNA, si siRNA.

ILCs were isolated from the lungs of naïve mouse and differentiated into ILC3s. Compared with control siRNA treatment, *Card11* siRNA treatment significantly reduced the number of IL-7R^+^RORγt^+^ and IL-17^+^ Card11^+^ cells in the IL-7R^+^RORγt^+^ population (Fig. 5a–c). We then evaluated the effect of Card11 inhibition on LPS-stimulated M1 macrophages in BMDMs derived from naïve mice. Similarly, compared with control siRNA treatment, *Card11* siRNA treatment significantly reduced the CD11b^+^Card11^+^ and Pro-IL-1β^+^ in the CD11b^+^Card11^+^ population in M1 macrophages (Fig. 5d, e).

## Discussion

On the basis of a cluster analysis of time-series gene expression data, we selected *Card11* as an important gene associated with AHR in HFD-induced obese mice that links adipose and lung tissues. Card11 expression was significantly increased in M1 macrophages (IL-1β^+^CD11c^+^CD206^–^ in CD11b^+^) of the adipose tissue and ILC3s (RORγt^+^ in IL7R^+^ of Lin^–^) of the lung tissue from HFD-induced obese mice. In addition, CARD11^+^ populations in ILC3s and LPS-stimulated IL-1β^+^CD16^+^ monocytes from the PBMCs of obese individuals with asthma were significantly greater than those of obese controls or nonobese individuals with asthma. Finally, we confirmed that AHR in HFD-induced obese mice disappeared when the Card11 signaling pathway was inhibited by the administration of *Card11* siRNA during the first or last 7 weeks of 13-week HFD feeding. We hypothesized that these findings may be due to the *Card11* siRNA-induced decrease in M1 macrophages in adipose tissue and ILC3s in lung tissue, as we observed in the in vitro experiments. Taken together, these findings indicate that Card11 contributes to the development of AHR in HFD-induced obese mice by affecting immune cells in both adipose and lung tissues. Interestingly, the effects of Card11 seem to be critical in the middle stage of HFD feeding. To the best of our knowledge, this is the first report demonstrating the potential role of Card11 in the development of obesity-associated AHR.

HFD-induced AHR in obese mice results from a complex interplay among many genes in multiple tissues. Therefore, clustering of time series gene expression data in adipose and lung tissues provides an opportunity to analyze such complex interactive biological phenomena. The dynamic changes in *Card11* expression in adipose and lung tissues suggest that Card11 may be an important molecule that mediates interactions between the two tissues, resulting in the development of AHR in HFD-induced obese mice. As mentioned previously, Card11 has been widely studied in inflammatory diseases including autoimmune disorders, and can induce inflammation mainly through the activation of T cells and the NF-κB pathway^12–14,29^. As mentioned previously, HFD-induced AHR was independent of adaptive immunity but dependent on ILC3 producing IL-17^4^. We confirmed that Card11 inhibition significantly reduced IL-17^+^RORγt^+^ in ILC3s differentiation from ILCs in naïve mouse lungs in vitro. RORγt is a Th17 cell-lineage transcription factor^34^. In adipose tissue, we focused on M1 macrophages, as macrophage-derived IL-1β production was induced by a HFD, and blockade of IL-1β with an IL-1 receptor antagonist abolished HFD-induced AHR^4^. In this study, we observed that Card11 inhibition significantly attenuated the induction of pro-IL-1β^+^ M1 macrophages by LPS from BMDMs in vitro. Overall, Card11 contributes to AHR development in HFD-induced obese mice by modulating immune cells in both adipose (M1 macrophages) and lung tissues (ILC3s). However, reduced activity of CARD11 promotes Th2 polarization, resulting in severe atopic dermatitis^35^. In addition, ~55% of patients with allergic asthma have hypomorphic dominant-negative *CARD11* mutations^14^. CARD11 likely has a different role in the pathogenesis of obesity-associated AHR than that in allergy-driven AHR.

One of the strengths of this study is that we evaluated the relevance of CARD11 in the pathogenesis of obesity-associated AHR using human samples. We recruited only nonatopic individuals to dissect obesity- and allergy-associated mechanisms for AHR and found that CARD11 expression was significantly greater in ILC3s and CD16^+^ monocytes from PBMCs of obese individuals with asthma than in those from obese controls or nonobese individuals with asthma. In previous reports, the number of CD16^+^ monocytes with very low expression of surface CD14, so-called nonclassical monocytes, increased under inflammatory conditions^36,37^. We observed that surface CD14 expression decreased upon LPS stimulation (data not shown). An increased CARD11^+^ population in LPS-stimulated IL-1β^+^CD16^+^ monocytes may be involved in obesity-associated AHR.

Another interesting finding of the present study was that the abrogation of HFD-induced AHR by Card11 signal inhibition was dependent on the time of *Card11* siRNA administration. The middle stage (6~7 weeks) of the 13-week HFD feeding period was critical. As shown in Fig. 2, the mean gene expression (MGE) of the adipose tissue gene Cluster 4 (containing *Card11*) in the HFD group increased as early as 4 weeks after HFD initiation and steadily increased, whereas that in the Chow group remained unchanged. Moreover, the MGE of the lung tissue gene Cluster 1 (containing *Card11*) did not differ between the HFD and Chow groups at 7 weeks after HFD initiation, but that in the HFD group abruptly increased and surpassed that in the Chow group at 10 weeks. The middle of HFD feeding is likely the point at which Card11-induced changes in adipose and lung tissues critically interact with the development of AHR. At 7 weeks after HFD initiation, HFD-induced mice presented a moderate increase in body weight (mildly obese) and did not exhibit AHR (Supplementary Fig. 1). Taken together, these findings suggest that a preventive strategy for obesity-associated AHR should be initiated, at least when moderate weight gain occurs. Our observations raise another important issue in understanding obesity-associated asthma. Although murine models of HFD-induced AHR are widely used, we should recognize that a HFD results in exceedingly overweight mice, which are not comparable to obese patients with asthma. Instead, it is possible that mild obesity (similar to that in mice at 7 weeks after HFD initiation in this study) confers susceptibility to nonspecific airway irritation, resulting in AHR development. Interestingly, mild obesity has been reported to be a risk factor for the development of various inflammatory diseases^38,39^, which supports our hypotheses.

In conclusion, we identified *Card11* gene as a key gene related to HFD-induced AHR in mice on the basis of a cluster analysis of time series gene expression of adipose and lung tissues. We confirmed the critical role of Card11 in the development of AHR by observing that AHR in HFD-induced obese mice was abolished by Card11 signal inhibition. These findings must be further validated with additional experimental studies.

## Supplementary information

Supplementary Information
