# Supplementary material for: Essential role of Card11 in airway hyperresponsiveness in high-fat diet-induced obese mice
Source: Exp Mol Med. 2024 Dec 13;56(12):2747–54. doi: 10.1038/s12276-024-01367-z (PMC11671588; doi:10.1038/s12276-024-01367-z)

SUPPLEMENTARY MATERIAL

Essential role of Card11 in airway hyperresponsiveness in high-fat diet-induced obese mice

Hyun-Seung Lee, PhD[1], Byung-Keun Kim, MD, PhD[2], Suh-Young Lee, MD, PhD[3], Hyuktae Kwon, MD, PhD[4], Heung-Woo Park, MD, PhD[3,5]

[1]Biomedical Research Institute, Seoul National University Hospital, Seoul, Republic of Korea;

[2]Department of Internal Medicine, Korea University College of Medicine, Seoul, Republic of Korea;

[3]Department of Internal Medicine, Seoul National University Hospital, Seoul, Republic of Korea;

[4]Department of Family Medicine, Seoul National University Hospital, Seoul, Republic of Korea;

[5]Department of Internal Medicine, Seoul National University College of Medicine, Seoul, Republic of Korea

METHODS

Phenotyping of control individuals and asthmatics

All were adults aged ≥ 19 years, non-smokers or former smokers with smoking pack years < 10, and non-atopic (negative by skin prick test with for inhalant allergens). The control group had no previous history of asthma and smoking, and normal lung function (predicted value of forced expiratory volume in one second [FEV1] ≥ 80% and FEV1/forced vital capacity ≥ 70%). The diagnosis of asthma was as follows: 1) presence of one or more of the following symptoms: dyspnea, cough, or wheezing, and 2) proven airway hyperresponsiveness (AHR) or airway reversibility. AHR was determined as positive when PC_{20} (provocative concentration causing a 20% fall in FEV1) was less than 16 mg/mL in a bronchial provocation test using methacholine chloride, and airway reversibility was defined as an increase in FEV1 ≥ 12% from baseline after inhalation of 400 μg of salbutamol or four weeks of anti-inflammatory treatment (systemic and inhaled corticosteroids).

SUPPLEMENTARY FIGURE LEGENDS

Supplementary Fig. 1. Evaluation of changes in HFD-induced obese mice at each time point

a Body weight and epididymal white fat tissue. b Methacholine (Mch)-induced airway hyperresponsiveness (Mch 100 mg/ml) in each group (Y-axis represents $cmH_2O/mL/s$). c-e Histological examination of the lung tissue (c, H&E stain, × 200; d, Masson's trichrome stain, × 400) and adipose tissue (e, H&E stain, × 200) in each group. All scale bars represent 100 µm. f, g Representative gaiting strategy and the frequency of neutrophil (Gr-1$^+$CD11c$^-$ in CD11b$^+$) from the lung tissue in each group. Five animals per group. Results are presented as mean ± standard deviation. #, differences in the HFD group ($^{\#}P < 0.05$ and $^{\#\#}P < 0.01$; compared to 4 weeks). §, differences in the Chow group ($^{\S}P < 0.05$ and $^{\S\S}P < 0.01$; compared to 4 weeks). *, differences between the HFD and Chow group ($^{*}P < 0.05$ and $^{**}P < 0.01$). Statistical analysis followed by two-way ANOVA with Sidak's multiple comparisons test. Chow, normal chow diet; HFD, high-fat diet.

Supplementary Fig. 2. The persistency of reduction in Card11$^+$CD45$^+$ cells in mouse lung cells after *Card11* siRNA administration

a, b Gating plot and the frequency of Card11$^+$CD45$^+$ in the lung cells from mice treated by control siRNA or *Card11* siRNA at each time point. The displayed percentage represents the population of Card11$^+$CD45$^+$ in total cells. Five animals per group. Results are presented as mean ± standard deviation. $^{\#\#}P < 0.01$ (control siRNA *vs. Card11* siRNA). Statistical analysis followed by two-way ANOVA with Sidak's multiple comparisons test. Si, siRNA.

Supplementary Fig. 3. Evaluation of changes treated by control siRNA in HFD-induced obese mice at each time point

a Experimental protocol. b Body weight and epididymal white fat weight in each group. c Methacholine (Mch)-induced AHR (Mch 100 mg/ml) in each group (Y-axis represents $cmH_2O/mL/s$). d The frequency of neutrophils ($Gr\text{-}1^+CD11c^-$ in $CD11b^+$) in the lung tissue. e The frequency of $CD127^+ROR\gamma t^+$ ILC3s in the lung tissue. f The frequency of M1s ($CD11c^+CD206^-$) in $CD11b^+$ cells in the adipose tissue. g, h Histological examination of mouse lung (g, H&E stain, × 200) and adipose tissue (h, H&E stain, × 200). Five animals per group. Results are presented as mean ± standard deviation. $^*P < 0.05$, $^{**}P < 0.01$ (compared to the Chow group). Statistical analysis was performed using one-way ANOVA with Bonferroni's multiple comparisons test. Chow, normal chow diet; HFD, high-fat diet; si, siRNA.

Supplementary Table 1. List of anti-mouse and anti-human antibodies for flow cytometry

Product	Company	Clone	Order number	Dilution factor
CARD11	Santa cruz	A-4	sc-166910 AF594	1:200
APC anti-mouse Gr-1	Biolegend	RB6-8C5	108411	1:100
FITC anti-mouse CD11c	Biolegend	N418	117306	1:100
FITC anti-mouse/human CD11b	Biolegend	M1/70	101206	1:100
FITC anti-mouse FceRIa	Biolegend	01-Mar	134305	1:100
FITC anti-mouse CD19	Biolegend	6D5	115506	1:100
CD49, FITC	Thermo	DX5	11-5971-82	1:100
CD3e, FITC	Thermo	145-2C11	11-0031-82	1:100
F4/80, FITC	Thermo	BM8	11-4801-85	1:200
BV711 anti-mouse CD4	Biolegend	RM4-5	100557	1:200
BV421 anti-mouse IL-17	Biolegend	TC11-18H10.1	506926	1:100
Rorγt, APC	Thermo	AFKJS-9	17-6988-80	1:100
PE/cy7 anti-mouse IL-7R	Biolegend	A7R34	135014	1:50
CD45, PerCP-Cy5.5	Thermo	30-F11	45-0451-80	1:200
APC/cy7 anti-mouse CD11c	BD	HL3	561241	1:100
Mouse CD206	R&D systems		AF2535	1:250
Pro-IL-1β, PE/cy7	Thermo	NJTEN3	25-7114-82	1:200
Alexa Flur 700 anti-human CD16	Biolegend	3G8	302026	1:50
FITC anti-human Lineage cocktail	Biolegend	UCHT1;HCD14;3G8;HIB19, 2H7;HCD56	348801	1:20
FITC anti-human CD11c	Biolegend	3.9	301604	1:50
FITC anti-human CD123	Biolegend	6H6	306014	1:50
PE/cy7 anti-human CD127	Biolegend	A019D5	351320	1:50

APC anti-human CD117	Biolegend	104D2	313206	1:50
BV 711 anti-humanCD336(NKp44)	BD Biosciences	p44-8	744303	1:50
PE anti-human RORγt	BD Biosciences	Q21-559	563081	1:100
FITC anti-human IL-1β	Biolegend	H1b-98	511705	1:100

Isotype antibody

Product	Company	Clone	Order number	Dilution factor
APC Rat IgG2b, κ Isotype Control	Biolegend	RTK4530	400611	1:1000
FITC Armenian Hamster IgG Isotype Control	Biolegend	HTK888	400905	1:1000
FITC Rat IgG2b, κ Isotype Control	Biolegend	RTK4530	400633	1:1000
Rat IgM Isotype Control, FITC	Thermo	eBRM	11-4341-82	1:1000
Brilliant Violet 421™ Rat IgG1, κ Isotype Control	Biolegend	RTK2071	400429	1:500
Rat IgG1 κ Isotype Control, APC	Thermo	eBR2a	17-4321-81	1:1000
PE/Cy7 Rat IgG2a, κ Isotype Control	Biolegend	RTK2758	400521	1:1000
APC-Cy™7 Hamster IgG1, λ1 Isotype Control	BD Biosciences	G235-2356	561206	1:1000
Donkey Anti-Goat IgG H&L (Alexa Fluor 647)	Abcam		Ab150131	1:1000
Rat IgG1 kappa Isotype Control, PE-Cyanine7	Thermo	eBRG1	25-4301-82	1:1000
FITC Mouse IgG2b, κ Isotype Control	Biolegend	MG2b-57	401205	1:1000
FITC Mouse IgG1, κ Isotype Control	Biolegend	MOPC-21	400107	1:1000
PE/Cy7 Mouse IgG1, κ Isotype Control	Biolegend	MOPC-21	400125	1:1000
APC Mouse IgG1, κ Isotype Control	Biolegend	MOPC-21	400119	1:1000

PE Mouse IgG2b, κ Isotype Control	BD Biosciences	27-35	555058	1:1000
FITC Mouse IgG2b, κ Isotype Control	Biolegend	MPC-11	400309	1:1000

Blank indicates polyclonal antibody

Supplementary Table 2. Membership genes belong to 4 clusters identified in clustering analysis of time-series gene expressions in adipose and lung tissue from HFD-induced obese mice

Adipose tissue	Membership genes
Cluster 1 (447 genes)	*Aacs; Abca5; Acacb; Acadsb; Ace2; Ackr4; Acsm3; Acsm5; Acss3; Acta1; Acvr1c; Adrb3; AI480526; Alad; Aldh1a1; Aldh2; Aldh5a1; Aldh6a1; Angpt1; Ankef1; Ankrd29; Aox3; Apbb3; Apcdd1; Apol11b; Apold1; Ar; Arhgap42; Arhgef28; Arhgef9; Arrdc2; Arrdc3; Art3; Ascl1; Asns; Atp10d; Atp2b4; Atp6v0e2; Auts2; B130011K05Rik; B230208H11Rik; B430319H21Rik; Bag4; Bbs2; BC035947; Bcam; Bcat2; Bche; Bckdha; Bckdhb; Birc2; Bmp4; Bnc2; Bphl; Btbd8; Btc; C2; C6; C7; C920006O11Rik; Ccbe1; Cd164l2; Cdc37l1; Cdo1; Cecr2; Cenpv; Ces1d; Ces1f; Cfd; Chchd6; Chdh; Chrdl1; Chrnb1; Cldn22; Clmn; Clpx; Clstn2; Cnr1; Cnst; Cobll1; Cp; Crebrf; Crls1; Csad; Cuedc1; Cyp27a1; Cyp2d22; Cyp2e1; Cytl1; D230025D16Rik; D3Ertd751e; D730048I06Rik; Dbt; Defb20; Defb48; Dennd2d; Dennd5b; Dgka; Dgkb; Dhtkd1; Dkc1; Dmrt2; Dnah3; Dnajb5; Dpyd; Drd1; Dusp16; Dusp18; Dysf; Ebf2; Echdc2; Echdc3; Echs1; Efcab7; Ehbp1; Eif4ebp3; Eml5; Enpp2; Ephb4; Ephx2; Epm2a; Ercc5; Eri2; Eva1c; F11r; Fam120aos; Fam120c; Fam132a; Fam13a; Fam150b; Fam180a; Fam188b; Fam214a; Fam35a; Fbxo31; Fbxo45; Fcamr; Fdx1; Fgf10; Fgfr2; Fgfr3; Fign; Firre; Fmo1; Fmo5; Foxc1; Fv1; Fyco1; Gabbr1; Gata6; Gdap10; Gde1; Ggnbp2os; Glb1l2; Golgb1; Gpat4; Gphn; Gpr165; Gpx3; Gpx5; Grhl1; Grhpr; Gsta4; Gstk1; Gstt1; Gulp1; Gzf1; H2-Ke6; H2-M9; Hey2; Hibadh; Hibch; Hnrnpu; Hoxa4; Hoxa5; Hoxa7; Hoxb2; Hoxc8; Hpgd; Hsd17b10; Hsf3; Iars2; Id1; Ift57; Ift88; Il17re; Inmt; Insr; Iqcb1; Irak1bp1; Isl1; Isoc2a; Itm2a; Jag2; Kalrn; Kat2b; Kcns3; Kctd15; Kctd17; Kdsr; Klhl11; Klhl2; Lcn8; Lcn9; Ldhb; Lekr1; Lncpint; Lnx1; LOC100862043; LOC102642832; Lrba; Lvrn; Lypd8; Lyplal1; Maob; Map3k5; Map3k7cl; Mapt; Mc5r; Mccc1; Mccc2; Med12l; Meis1; Meis2; Met; Mettl20; Mettl7a1; Mgst1; Mical3; Micu3; Mir130b; Mir1898; Mir194-2; Mir3074-2; Mir32; Mir383; Mir466h; Mir872; Mir99a; Mira; Mrgprh; Mrps34; Muc16; Myh10; Myh11; Myo1b; Nampt; Ncoa1; Ndrg1; Ndufa6; Ndufb11; Negr1; Neo1; Net1; Notch1; Nova1; Nr1d1; Nrbp2; Nrg4; Oard1; Odf2l; Olfr1015; Olfr1033; Olfr1100; Olfr1158; Olfr1448; Olfr175-ps1; Olfr198; Olfr235; Olfr498; Olfr550; Olfr875; Opn3; Orm3; Osbpl11; Oscp1; Osr1; Pabpc6; Pacsin3; Paqr3; Park2; Pck1; Pcsk6; Pde1a; Pde3b; Pdk2; Pdpk1; Peli2; Penk; Per3; Pex3; Pfkfb1; Phc1; Phf2; Phkg1; Phpt1; Phyh; Phyhd1; Pi15; Pkhd1l1; Pla2g12a; Plagl1; Platr14; Plpp6; Pm20d2; Pmvk; Pnisr; Podn; Polr3gl; Pon1; Porcn; Ppa1; Ppl; Ppp2r3a; Ppp4r4; Prdx6; Prex2; Prkd1; Prodh; Prrg3; Pxmp2; Pygl; Rad9b; Ralgapa2; Ranbp9; Rasl11b; Rassf6; Raver2; Rbm4b; Rbp7; Reep6; Reln; Retn; Rev1; Rgma; Rgs7; Rhbdd3; Rhbdl1; Rhobtb1; Rhou; Ripk4; Rnpc3; S100a1; Scara5; Scarna3a; Sccpdh; Scn7a; Scp2; Sctr; Sdr39u1; Sema3c; Sema5a; Sfxn1; Sh3d21; Sik3; Slc1a1; Slc1a3; Slc20a2; Slc25a23; Slc25a35; Slc25a44; Slc2a3; Slc38a5; Slc43a1; Slc44a3; Smim1; Snhg12; Snora15; Snora17; Snora28; Snora30; Snora34; Snora44; Snora52; Snora69; Snord4a; Snord59a; Snord66; Snord90; Sntg2; Sorcs1; Sort1; Sox6os; Spata22; Sqrdl; Sra1; St6galnac5; Steap2; Stim1; Stx1b; Suclg2; Sugct; Sult1e1; Tcaim;*

	Tead1; Tef; Thap6; Thrsp; Timm21; Timp4; Tle1; Tmeff1; Tmem134; Tmem147os; Tmem223; Tmem56; Tmem88b; Tmtc1; Tpmt; Trbv12-2; Trit1; Trpc1; Tshz2; Tst; Ttc21b; Ttc8; Txlng; Uimc1; Upk1b; Urb1; Usp53; Usp54; Vegfa; Vit; Vmn2r44; Vnn1; Vnn3; Vps13a; Wdr35; Wdsub1; Yes1; Zbtb10; Zbtb11os1; Zbtb20; Zc3h7b; Zfand1; Zfhx4; Zfp462; Zfp507; Zfp512b; Zfp609; Zfp809; Zhx2; Znfx1; Zrsr1; Zscan4e; Zyg11a

Cluster 2 (767 genes)	*AB124611; Abcb4; Abcg1; Abhd12; Acer3; Actn1; Actr3; Adam17; Adam19; Adam22; Adam8; Adcy3; Adcy7; Adgre1; Adssl1; AF251705; Ahnak2; AI413582; AI662270; AI839979; Aim2; Akr1b8; Alcam; Aldh3b1; Alox5ap; Alpk1; Ankdd1a; Anpep; Anxa4; Ap2m1; Apaf1; Aph1c; Apobec1; Aprt; Arap1; Arhgap10; Arhgap11a; Arhgap17; Arhgap19; Arhgap22; Arhgap25; Arhgap30; Arhgap6; Arhgap9; Arhgdib; Arhgef6; Arl11; Arntl; Arpc5; Arrb2; As3mt; Asah1; Asb4; Asf1b; Aspm; Atad2; Atf3; Atp13a2; Atp1a3; Atp6ap2; Atp6v1a; Atp6v1b2; Atp6v1c1; Atp8b4; AU020206; AU022793; B4galt5; B4galt6; B4galt7; Bax; BC005537; BC028528; BC055324; Bcl2a1b; Bcl2a1d; BE692007; Bin2; Birc5; Blm; Blnk; Blvra; Bmper; Brca1; Brip1; Bst1; Btk; Bub1; Bub1b; C1qa; C1qb; C1qc; C330027C09Rik; C3ar1; C430042M11Rik; C5ar1; C5ar2; C920009B18Rik; Cacna1a; Calhm2; Camk1d; Capg; Capza1; Casc5; Casp1; Casp3; Cass4; Cbfb; Cbl; Cbr3; Ccdc88a; Ccl3; Ccl5; Ccna2; Ccnb1; Ccnb2; Ccr3; Ccr5; Ccrl2; Cd180; Cd200r1; Cd200r4; Cd22; Cd300a; Cd300lb; Cd300ld; Cd37; Cd44; Cd48; Cd53; Cd68; Cd72; Cd83; Cd84; Cd9; Cdc42se1; Cdc42se2; Cdca5; Cdca7l; Cdca8; Cdk1; Cdk15; Cdk18; Cdk20; Cdkn3; Cdt1; Ceacam16; Cenpa; Cenpe; Cenpf; Cenpi; Cenpl; Cenpn; Cep290; Cep55; Cerk; Ckap2; Ckap4; Ckb; Clec12a; Clec4a1; Clec4a2; Clec4a3; Clec4d; Clec5a; Clec7a; Clic1; Cln3; Cma1; Cmtm3; Cmtm7; Cnr2; Colgalt1; Coro1a; Coro1c; Cotl1; Cpa3; Cpe; Cpxm1; Creg1; Csf1r; Csf2ra; Csf2rb2; Csk; Cstb; Ctsa; Ctsh; Ctsk; Ctsl; Ctss; Cxcl14; Cxcl16; Cxcr4; Cyb5r4; Cyba; Cybb; Cyfip1; Cyfip2; Cyth4; D330050G23Rik; Daglb; Dapp1; Dbf4; Dck; Dclre1c; Dcstamp; Ddias; Dennd4b; Dennd4c; Depdc1a; Dgkz; Dhrs9; Diaph3; Dlg3; Dlgap5; Dnase1l1; Dnmt1; Dnmt3a; Dnmt3aos; Dock10; Dock2; Dok1; Dpep2; Dpp7; Dpy19l3; Dtx4; Dusp7; E2f8; Ebi3; Ect2; Efhd2; Efr3b; Ehd4; Eid3; Elmo1; Elmo2; Emp3; Eno2; Entpd1; Eps8; Esco2; Evi2a; Evl; Eya4; F10; F7; Fabp7; Fam105a; Fam111a; Fam122b; Fam198b; Fam83f; Fam96a; Fat3; Fblim1; Fcer1a; Fcer1g; Fcgr1; Fcgr3; Fcgr4; Fermt3; Fgd3; Fgd4; Fgd6; Fgf7; Fhl2; Fignl1; Fmn1; Fnip2; Fos; Frmd4a; Frmd4b; Frrs1; Ftl1; Fundc1; Fxyd5; Fyb; Gabra3; Galc; Galns; Galnt6; Galnt7; Gas2l3; Gas7; Gatm; Gdf3; Gdpd1; Gga3; Gins1; Gla; Glipr1; Glrx; Gmip; Gmnn; Gna13; Gnpda1; Got1; Gpm6b; Gpr137b; Gpr137b-ps; Gpr176; Gpr183; Gpr34; Gpr50; Gpr65; Gpsm3; Grn; Gsap; Gusb; H2-M2; H2-T23; Hacd4; Havcr2; Hck; Hcls1; Heatr5a; Hells; Hexa; Hexb; Hgf; Hilpda; Hist1h1b; Hist1h2ag; Hist1h2bg; Hist1h4i; Hist1h4m; Hist2h2ab; Hist2h2ac; Hk3; Hmga1-rs1; Hmmr; Hmox1; Hpcal1; Hpgds; Hpse; Hsph1; Htr2b; Id2; Ifi30; Ifnar1; Igf2bp2; Igf2r; Igkv3-2; Igsf6; Ikbke; Ikzf1; Il10ra; Il10rb; Il1rn; Il27ra; Il7r; Incenp; Inpp5d; Iqgap1; Irf5; Irf8; Itga4; Itgad; Itgam; Itgav; Itgax; Itgb2; Itsn1; Kcnab2; Kcnj2; Kcnk6; Kcnn4; Kif11; Kif15; Kif20a; Kif20b; Kif23; Kif3b; Klhl6; Knstrn; Kntc1; Lacc1; Lair1; Laptm5; Lat2; Layn; Lcp2; Ldlrap1; Lgmn; Lig1; Lilr4b; Lilrb4a; Lin9; Lipa; LOC100503338; LOC102638993; Lpar5; Lpxn; Lrmp; Lrp12; Lrrc25; Lst1; Lum; Ly86; Ly9; Ly96; Lyn; Lyz2; Mad2l1; Madd; Mafb; Mamdc2; Mamld1; Man1c1; Man2b1; Manba; Maoa; Map4k1; Mastl; Mb21d1; Mcm3; Mcm4; Mcm5; Mcm6; Mcoln2; Mcpt4; Melk; Metrnl; Mfsd1; Mfsd11; Mfsd12; Mfsd7b; Mgat2; Mgat5; Milr1; Mir142hg; Mir342; Mir669n; Mis18bp1; Mitf; Mki67; Mkl1; Mmp12; Mmp13; Mmp14; Mmp19; Mmp2; Mmp3; Mocos; Mpeg1; Mrgprb1; Mrgpre; Ms4a14; Ms4a4b; Ms4a6c; Ms4a6d; Ms4a7; Msr1; Mtrf1; Mtss1; Muc13; Mup1; Mup13; Mup14; Mup15; Mup16; Mup18; Mup19; Mup2; Mup7; Mup8; Mybl1; Myd88; Myh15; Myo1e; Myo1f; Myo1g; Myo9b; Myof; Mypopos; Naip6; Ncapg; Ncaph; Nceh1; Ncf1; Ncf4; Nckap1l; Ncs1; Ndc80; Necap2; Neurl3; Nfam1; Nfasc; Nfkbid; Nfya; Nipa2; Nlrc3; Nlrc4; Nostrin; Npas2; Npc1; n-R5s26; Nrcam; Nrp2; Nuak2; Nuf2; Nusap1; Nutf2; Oas1g; Oasl1; Oit3; Olfr110; Olfr111; Olr1; Osbpl3; Osbpl8; P2rx4; P2rx7; P2ry14; P2ry6;*

	Panx1; Pbk; Pcna; Pcolce; Peak1os; Phf20; Pi4k2a; Pianp; Pik3ap1; Pik3cd; Pik3cg; Pik3r5; Pip4k2a; Pirb; Pitpnc1; Pkib; Pla2g15; Pla2g7; Plagl2; Plau; Pld3; Plek; Plekhm2; Plekhm3; Plekhn1; Plekho1; Plekho2; Plin2; Plk1; Plk2; Plk4; Pltp; Plxna1; Plxnb2; Plxnc1; Pole; Pole2; Polh; Prc1; Prcp; Prex1; Prim1; Prim2; Prkcb; Prkcd; Prr11; Prune2; Psd4; Pstpip1; Ptafr; Ptchd1; Ptgfrn; Ptgr1; Ptpn6; Ptpn7; Ptprc; Ptpro; Qpct; Rab11fip5; Rab31; Rab3il1; Rab7b; Rab8b; Rac2; Racgap1; Rasa1; Rasa4; Rasal3; Rassf3; Rassf4; Rassf8; Rbpsuh-rs3; Rel; Renbp; Reps2; Rgs1; Rgs10; Rgs18; Rhog; Rhoh; Rnaseh2b; Rnh1; Ror1; Rpf2; Rps6ka1; Rps6ka2; Rrm1; Rrm2; Rufy3; S100a4; S1pr2; Sap30; Sash3; Sass6; Scamp5; Scpep1; Sdc3; Sdcbp; Selplg; Serpinb6b; Serpinb9b; Sh2d1b1; Sh3bgrl3; Sh3bp2; Sh3pxd2b; Shcbp1; Shisa5; Shisa6; Shtn1; Siglec1; Sirpa; Sirpb1a; Sirpb1b; Ska3; Skap2; Sla; Slamf7; Slamf8; Slc11a1; Slc15a3; Slc15a4; Slc18a1; Slc25a12; Slc25a24; Slc25a43; Slc35f6; Slc37a2; Slc38a1; Slc39a10; Slc40a1; Slc43a2; Slc6a6; Slc6a8; Slc7a7; Slc9a4; Slc9a9; Slco2b1; Slfn2; Slfn9; Smap2; Smc2; Sned1; Snord19; Snord96a; Snx20; Snx24; Snx27; Snx30; Snx5; Snx8; Soat1; Spag5; Spc25; Spdl1; Specc1; Spi1; Spred1; Srxn1; Ssc5d; St18; St8sia4; Stab1; Stac2; Stard3; Stard8; Steap3; Stmn1; Stra6l; Stxbp3; Syk; Syngr1; Tacc3; Tagln2; Taok3; Tbxas1; Tcirg1; Tfec; Tfr2; Tgfb1; Tgif1; Themis2; Ticam2; Ticrr; Tifa; Tifab; Timp1; Tk1; Tlr1; Tlr13; Tlr2; Tlr6; Tlr7; Tlr8; Tm4sf19; Tm6sf1; Tmem104; Tmem106a; Tmem165; Tmem173; Tmem202; Tmem206; Tmod1; Tnf; Tnfaip2; Tnfaip8l2; Tnfrsf11a; Tnfrsf1b; Tnfrsf26; Tnip3; Tns3; Top2a; Tpcn2; Tpra1; Tpsb2; Tpx2; Trem2; Trerf1; Ttk; Tuba1c; Tubb5; Tubb6; Txnrd1; Tyrobp; Uap1l1; Ubash3b; Ube2c; Ucp2; Uhrf1; Ulbp1; Unc93b1; Vamp7; Vat1; Vav1; Vav3; Vipas39; Vsig4; Vsig8; Was; Wdfy4; Wdr1; Wdr91; Wee1; Wfs1; Wrb; Xylt1; Zc3h12d; Zdhhc14; Zfp273; Zfp82; Zfp850; Znf41-ps; Zranb3; Zwilch

Cluster 3 (365 genes)	*Abhd15; Abhd3; Acaca; Acad11; Acat1; Acot4; Adamts1; Adamts9; Adhfe1; Adig; Adipoq; Adipor2; Adnp2; Adrb2; AF357399; Ager; Agpat9; Agt; Ahcyl2; AK157302; Akap7; Alas2; Aldh1l1; Aldh7a1; Amd1; Amd2; Amy1; Amy2a5; Aox1; Apoc1; Appbp2; Arl4a; Arl4d; Atl2; Atp1a2; Atp5l; Atraid; Atxn7; B3galt2; B430010I23Rik; B930094E09Rik; Bag1; BB123696; Bmp2; Bnip3; Brpf1; Brpf3; C030006K11Rik; C730002L08Rik; Car1; Car5b; Caskin2; Ccbl2; Ccdc69; Ccdc71l; Ccnj; Cdh23; Cdk5rap1; Cebpd; Chd7; Chpt1; Chst1; Cisd1; Clic5; Clpb; Clpp; Cluh; Cnnm2; Cpeb2; Cry2; Csta1; Ctdspl; Cyb5a; Cyp2f2; Cypt8; D10Jhu81e; Dclre1b; Dcst1; Dcst2; Dctn3; Ddit4; Ddrgk1; Ddt; Dennd3; Dgat1; Dhdh; Disp2; Dnajc21; Drap1; Dtna; Dusp8; E030003E18Rik; Eci1; Eci3; Eepd1; Ehhadh; Eif4ebp1; Eif4ebp2; Elane; Epas1; Epha2; Exd1; Fah; Fam101b; Fam160a1; Fam195a; Fam204a; Fasn; Fbxl4; Fbxo21; Fermt2; Ffar4; Fgf2; Fhl5; Fitm2; Fkbp5; Flad1; Flt1; Foxo1; Frrs1l; Ganc; Gatb; Gbe1; Gcgr; Glt28d2; Gnai1; Gnb5; Gpam; Gpd2; Gprc5a; Gpt2; Grem2; Gsta3; Gstz1; Gys1; H1fx; H6pd; Hacl1; Hbb-bh1; Hcrtr2; Hdx; Hecw2; Heph; Hif3a; Hint2; Hk2; Hmces; Hoxc4; Hsd11b1; Hspb8; Igf2; Igkv12-47; Il15ra; Il17rc; Immp2l; Inafm2; Inca1; Irs1; Isca1; Itgb1bp1; Ivd; Kcnj15; Kctd2; Klb; Klf15; Klf9; Klhdc8b; Krtap31-2; Lamp3; Larp1b; Larp4; Lgals12; Lin52; Lipe; LOC102636563; LOC105243148; LOC105243404; LOC105244007; Lonp2; Lpin1; Lppos; Lrfn4; Lrig1; Lzts2; Mall; Map3k6; Map4k2; Map6d1; Mapk6; Mbd1; Mcc; Mgst3; Micall1; Mir215; Mir218-2; Mir26a-1; Mir3067; Mir694; Mknk2; Mlxipl; Mmp9; Mocs1; Mogat1; Mpst; Mut; Myrip; Ncam2; Ndufb8; Ndufs2; Ndufv1; Nfia; Nkain4; Nmb; Nnat; Nod2; Nos3; Nova2; Npdc1; Npr2; Nr1i3; Nrip1; Nts; Nudt12; Nudt18; Ogn; Olfr1026; Olfr1232; Olfr1263; Olfr1431; Olfr693; Oplah; Osgepl1; Ovgp1; P2rx1; Palm; Pccb; Pcx; Pdha1; Pdzd2; Pdzrn3; Peg13; Peli3; Pex11a; Pfkfb3; Pgm2; Phldb1; Phrf1; Phykpl; Pkdcc; Plekhf1; Plin4; Pln; Plppr3; Plxna2; Pm20d1; Pnpla3; Poldip2; Por; Ppara; Ppm1m; Ppp1r1a; Ppp1r3c; Ppp1r3e; Ppp1r9a; Ppp2r1b; Ppp2r2d; Prkaa2; Prlr; Proca1; Psg29; Ptp4a1; Ptpn11; Ptpn14; Ptpn4; Ptprd; Ptprm; Pygb; Pygo1; Rab34; Rapgef1; Rapgef3; Rasa2; Rbms2; Rbp4; Rcl1; Retnla; Retsat; Rgs7bp; Rnf144b; Rpl27a-ps2; Rufy4; Rxra; Samm50; Scgb1a1; Scn3b; Sdhaf1; Sema3a; Sema4c; Serpina3b; Serpina3j; Setd1a; Sft2d3; Sftpa1; Sftpc; Sh2b2; Sh3glb2; Sh3pxd2a; Siglech; Sik2; Slc22a23; Slc25a16; Slc25a33; Slc2a12; Slc2a13; Slc2a4; Slc35e4; Slc5a6; Slc6a4; Smim19; Smkr-ps; Smoc1; Smok3c; Snhg11; Snrk; Snta1; Sntb1; Sorbs1; Sowahc; Sox5it; Sox6; Sox7; Stc1; Stfa2l1; Syde2; Syn2; Syt14; Tab3; Taf9b; Tagln; Tcf15; Tead4; Thbd; Thra; Thrb; Thumpd1; Timd4; Tiparp; Tmem143; Tmem243; Tns1; Tomm6os; Trap1; Trav7d-5; Trim32; Tspo; Txnrd3; Ube2o; Uck1; Vegfb; Vmn1r67; Vprbp; Zcchc14; Zfand3; Zfp503; Zswim7*
Cluster 4 (233 genes)	*Abr; Adh6b; Aldh18a1; Aldh1a2; Ammecr1; Angptl1; Ap1s2; Ap1s3; Aplnr; Apol8; Arhgap39; Atp1a1; Atp6v0a1; Atp6v0d2; B430212C06Rik; B4galnt1; Bcar3; C2cd2; Cacna1d; Cadm1;* ***Card11****; Casq1; Ccdc18; Cck; Ccl2; Ccl7; Ccl8; Ccnd1; Cd300lf; Cd52; Cdc25c; Cdca3; Cdk6; Cdkl4; Cfi; Ciita; Cilp; Cit; Cklf; Clec10a; Clec4b1; Clspn; Col12a1; Col1a1; Col6a1; Col6a2; Col6a3; Cpt1a; Cysltr1; Dab2; Dcbld2; Dclk1; Ddah1; Dhcr7; Dna2; Dock8; Dot1l; Dppa4; Dse; Dtl; Duoxa1; Ear2; Egr2; Emb; Entpd7; Ephb2; Eya1; F2r; Fam134b; Fam20c; Fam26f; Fam83d; Fanci; Fen1; Ffar2; Fgf13; Fgr; Fhdc1; Fkbp1b; Frk; Gk; Gltp; Gpnmb; Gpr35; Grid1; Gtse1; H2-Aa; H2-Ab1; H2-DMa; H2-DMb1; H2-Eb1; Hdac9; Hephl1; Hist1h2ab; Hist1h2af; Hist1h2an; Hist1h2bf; Hist1h2bn; Hist1h3b; Hist1h4n; Hist2h3c2; Hmgcs1; Hs6st1; I830077J02Rik; Ifngr2; Il13ra1; Il13ra2; Iqgap3; Itgbl1; Itpk1; Kdelr3; Kif21b; Kif2c; Kit; Krt79; Lbh; Lcp1; Lctl; Lgals3; Lipf; Lonrf3; Malt1; Mapkapk3; Mcm10; Mcm2; Mfap4;*

	Micall2; Mir1901; Mkl2; Mrc2; Ms4a4c; Mstn; Naip2; Ncan; Ncf2; Nedd4l; Nfkb2; Nipal3; Noct; Npl; Nr2c2ap; Nt5dc2; Nup210; Optc; Otop1; Pak1; Pam; Paqr9; Parp8; Pcolce2; Pde7a; Pde7b; Pdxk; Pfkfb4; Phospho1; Pip5k1c; Plcg2; Plcl2; Pld4; Plod1; Plppr4; Plxdc1; Pmepa1; Postn; Prps1; Psmb10; Ptpn1; Ptpn22; Ptpre; Ptprj; Pvrl3; Rab29; Rab43; Rad54b; Rasgef1b; Rasl12; Reep4; Rell1; Rnase2a; Rnf122; Rnf128; Rragd; Runx1; Ryr2; S100a8; Saa3; Sel1l3; Sgms2; Skp2; Slamf9; Slc25a13; Slc41a2; Slc5a7; Slc9a3r1; Sox4; Spp1; Spry3; Sptlc3; Srgn; St14; Stambpl1; Stap1; Syngr2; Tcf19; Tcl1b2; Tec; Tfpi2; Tjp2; Tldc1; Tlr9; Tmeff2; Tmem144; Tmem164; Tmem176a; Tmem229b; Tmem86a; Tmem97; Tnfrsf21; Tnfrsf23; Tnip1; Tpd52; Tpm3; Trac; Traj44; Trdn; Trim36; Trp53; Ttyh3; Tubb2a; Ubd; Unc79; Wfdc21; Zmiz1

**Lung tissue**	**Membership genes**
Cluster 1 (9 genes)	*Adam7; Adgre4; **Card11**; Fbxw15; Gpx5; Hspa1b; Plcb1; Snora36b; Vsig1*
Cluster 2 (81 genes)	*Adamts9; Akap12; Ampd3; Arntl; Arrdc2; Asns; Axdnd1; Bcar3; Ccnjl; Cdkn1a; Cfd; Ch25h; Clec4d; Cpne8; Creb5; Crem; Crispld2; Csf3; Cyp1a1; Cyp2e1; Dennd4a; F3; Fam124b; Fam160a1; Fpgs; Gadd45g; Gclc; Gfpt2; Gpr157; Gpr50; Hpx; Il1r2; Inhbe; Kcnj8; Lcn2; Lmcd1; LOC105243073; Magea10; Mat1a; Mfsd2a; Miat; Mir5133; Mir877; Mmp8; Mthfd2; Nlrp12; Npas2; Nup210l; Olfr1195; Olfr286; Olfr786; Ovgp1; Pde4d; Pi15; Poteg; Prl7c1; Prss12; Rasd1; Rasgrp3; Rdh12; Retnlg; Rhoj; Rnls; Rps15; Rxrg; Sema6b; Serpina3f; Serpina3h; Slc38a4; Slc7a5; Slc9b2; Stk32a; Stk-ps1; Tcf15; Tmem59l; Tnfaip6; Trav12-2; Trpm2; Urb1; Vmn1r6; Zglp1*
Cluster 3 (14 genes)	*Card11; Cr2; Dbp; Mir130a; Olfr1271; Olfr1342; Olfr555; Olfr675; Prdm8; Sh3rf2; Supt4b; Tnfsf10; Tspan4; Zscan4b*
Cluster 4 (10 genes)	*Bcas3os1; C5ar1; Cd300lf; Galnt15; Iigp1; Itih4; Lrtm2; Psg18; Samd5; Slc40a1*

The *Card11* gene (in red) belongs to Cluster 4 identified from adipose tissue and to Cluster 1 identified from lung tissue as well.

Supplementary Table 3. Functionally important genes belong to 4 clusters identified from lung tissue

Lung tissue gene cluster	Gene	Phenotypes directly attributed to mutations of this gene	Adipose tissue gene cluster
1	*Card11*	Abnormal B & T cell proliferation/differentiation<br>Decreased circulating IL-4 & IL-5 levels<br>Lung inflammation<br>Decreased airway responsiveness	4
	Gpx5	Decreased eosinophils	1
	Hspa1b	Increased IL-6 secretion	Not assigned
3	*Cr2*	Decreased neutrophils & B cells<br>Decreased immunoglobulin level	Not assigned
	Prdm8	Increased airway resistance	Not assigned
	Tnfsf10	Abnormal negative T cell selection,<br>Impaired natural killer cell mediated cytotoxicity	Not assigned
4	*C5ar1*	Impaired neutrophil recruitment<br>Abnormal humoral immune response	2
	Cd300lf	Abnormal mast cell physiology<br>Increased IL-4, IL-5 & IL-13 secretion<br>Respiratory system inflammation	4
	Slc40a1	Increased splenocyte apoptosis	2

Supplementary Table 4. Baseline demographics of human subjects enrolled

	Non-obese controls	Obese controls	Non-obese asthmatics	Obese asthmatics
	N = 10	N = 11	N = 10	N = 10
Age, year	53.5 ± 13.6	41.4 ± 10.6	53.5 ± 15.5	58.7 ± 13.9
Male, N	1	4	2	4
Body mass index, kg/m^2	21.6 ± 1.0	28.6 ± 2.3	21.1 ± 2.2	27.4 ± 2.6

Data are presented as mean ± standard deviation. N; number.

Supplementary Figure 1.

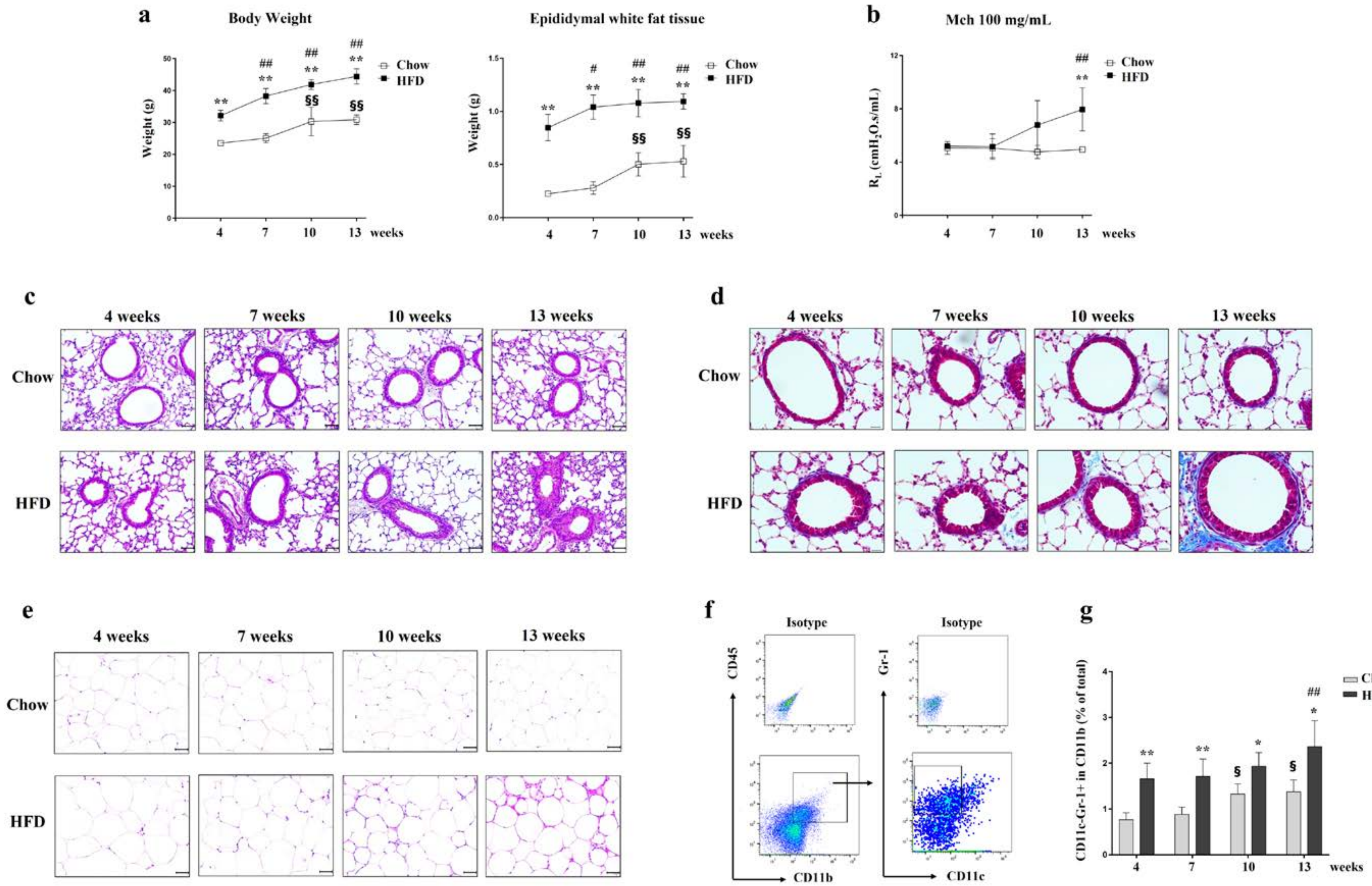

Supplementary Figure 2.

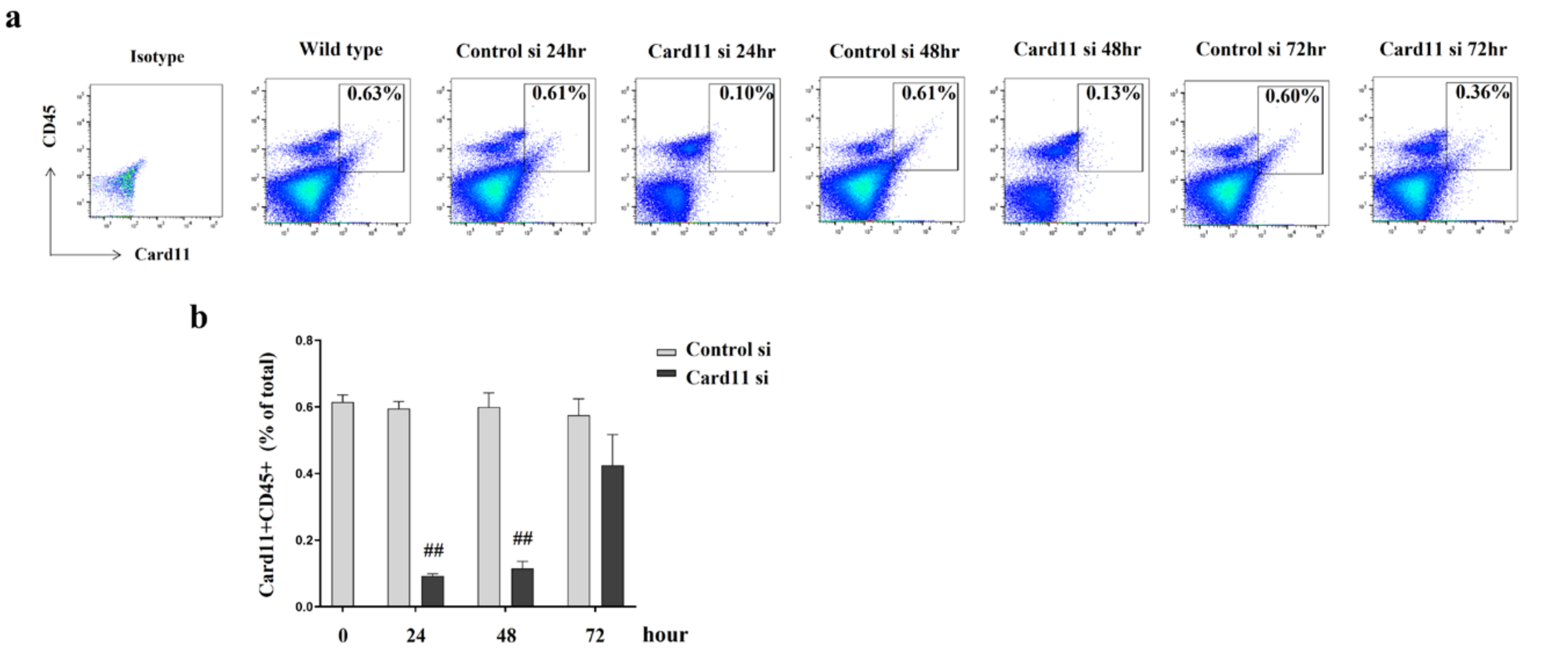

Supplementary Figure 3.

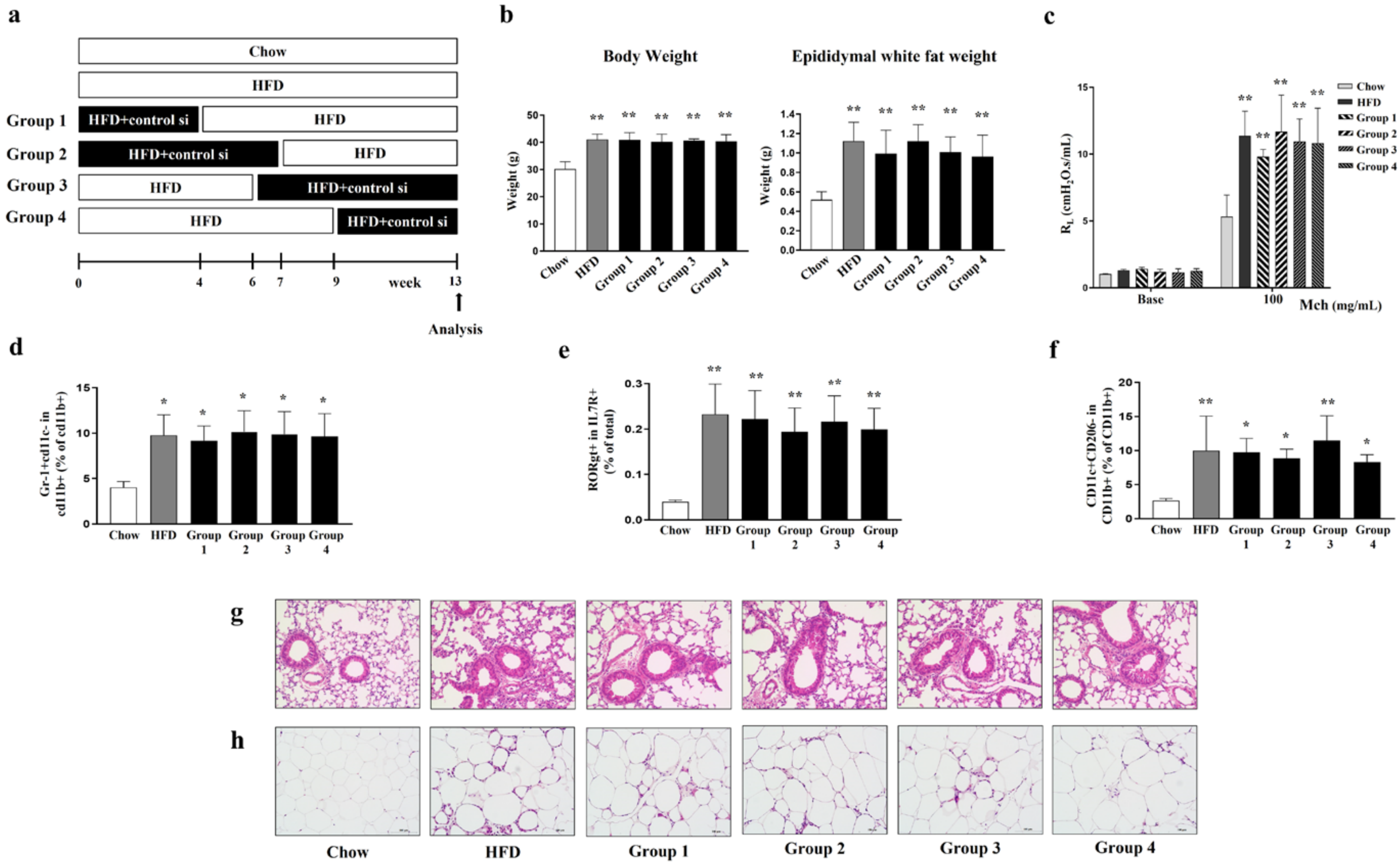

Supplement: Supplementary file 1 — Supplementary Information [file 12276_2024_1367_MOESM1_ESM.pdf]
